# The Working Hours of Private Nurses

**Published:** 1919-06-07

**Authors:** Gladys M. E. Leigh


### THE EDITOR'S LETTER-BOX.

### The Working Hours of Private Nurses.

*To the Editor of* The Hospital.

Sir,—The correspondent "A. H. I.," in her letter "Re Working Hours of Private Nurses," drops her subject at the identical spot where she commenced her discussion.

When "A. H. I." states "that the lines of a private nurse run on a parallel to that of a medical man," she voices a popular fallacy. A doctor charges a separate fee for each visit he pays, and between the hours of 10 p.m. and 8 a.m. his fees are doubled, he would indignantly repudiate any suggestion as to an inclusive charge.

If there can be "no solution for the problems of hours and the settlement of conditions for the nurse by rule or dogma," why do private-nursing institutions still perpetuate the farce of sending a copy of their rules to the relatives of each patient?

Also if during past years it has been found impossible to educate the public to an appreciation of nursing services apart from £ s.d., how does "A. H. I." hope to accomplish this miracle in the future?

In other words, this unfortunate branch of the nursing Profession is still to be unorganised, with no coherent voice, free for exploitation by possibly unscrupulous employers. Yours faithfully

Gladys M. E. Leigh.

[Miss Leigh overlooks the points of "A. H. I.'s" letter in the above reply. These points relate to the working hours, and not to the monetary compensation or the exploitation of the nurse.—Ed. The Hospital.]

